# Nursing of forearm hematoma after transradial coronary intervention

**DOI:** 10.1002/hsr2.70050

**Published:** 2024-08-30

**Authors:** Guangshuo Zhi, Mengjie Lei, Shuang Qian, Chunyan Zhang, Yachao Li, Zhigang Zhao, Zengming Xue

**Affiliations:** ^1^ Department of Cardiology, Langfang People's Hospital Hebei Medical University Langfang, No. 37, Xinhua Road China; ^2^ Department of Cardiology, Langfang People's Hospital Hebei Medical University, Langfang Core Laboratory of Precision Treatment of CAD Langfang, No. 37, Xinhua Road China

**Keywords:** case series, forearm hematoma, nursing, transradial coronary intervention

## Abstract

**Background:**

Complications such as forearm hematoma after coronary intervention through the radial artery are a common complication.

**Material and methods:**

By observing, describing, and analyzing the pictures taken during clinical diagnosis and consultation, we summarize the prevention, treatment, and nursing of forearm hematoma after percutaneous coronary intervention, to provide reference for the nursing of patients with forearm hematoma.

**Results:**

We have innovatively summarized the risk classification of forearm hematoma and the three key time points for preventing hematoma.

**Conclusion:**

Complications such as forearm hematoma after coronary intervention through the radial artery are a common complication. We have innovatively summarized the risk classification of forearm hematoma and the three key time points for preventing hematoma, providing reference for the prevention and management of forearm hematoma in clinical practice. For patients undergoing transradial coronary intervention, the three key time points for preventing hematoma and symptomatic management based the risk classification of forearm hematoma are crucial.

## Highlights

- By identifying risk factors and mastering management measures, the incidence of forearm hematoma after intervention can be effectively reduced, and patient comfort can be improved.
- Strengthening preoperative evaluation, standardized intraoperative procedures, postoperative care, and rational drug treatment are key measures for prevention.
- In the future, intelligent monitoring devices can be used for real‐time monitoring and pressure adjustment of forearm hematoma to improve the timeliness and accuracy of hematoma observation.

## 1 INTRODUCTION

With the continuous development of intervention technology, percutaneous coronary intervention (PCI) through the radial artery approach has become the preferred recommendation for coronary intervention due to its advantages of small trauma, convenient bandaging, fewer complications at the puncture site, no position limitation, and less patient pain.^1^ The 2018 European Heart Association guidelines for revascularization also recommend the radial artery approach as the standard approach.^2^ At present, the proportion of transradial coronary intervention in China exceeds 80%.^3^ The most common complication is forearm hematoma.^1^ Although forearm hematoma is the most common complication of PCI, the incidence of forearm hematoma is still low in most medical institutions except for those that have newly developed PCI, making it difficult to conduct large‐scale studies on forearm hematoma. However, forearm hematoma poses significant risks to patients, including nerve damage and tissue necrosis caused by compression of blood vessels and nerves. Therefore, this study summarizes our nursing experience for patients with forearm hematoma since the implementation of PCI, and demonstrates through “case” the good prognosis of using our summarized nursing experience to provide corresponding care for patients with forearm hematoma. This to some extent confirms the effectiveness of the nursing measures we have summarized, providing clinical reference for the nursing of patients with forearm hematoma.

## 2 MATERIAL AND METHODS

### 2.1 Statistics on incidence rate of forearm hematoma from 2017 to 2022

**Figure 1:**
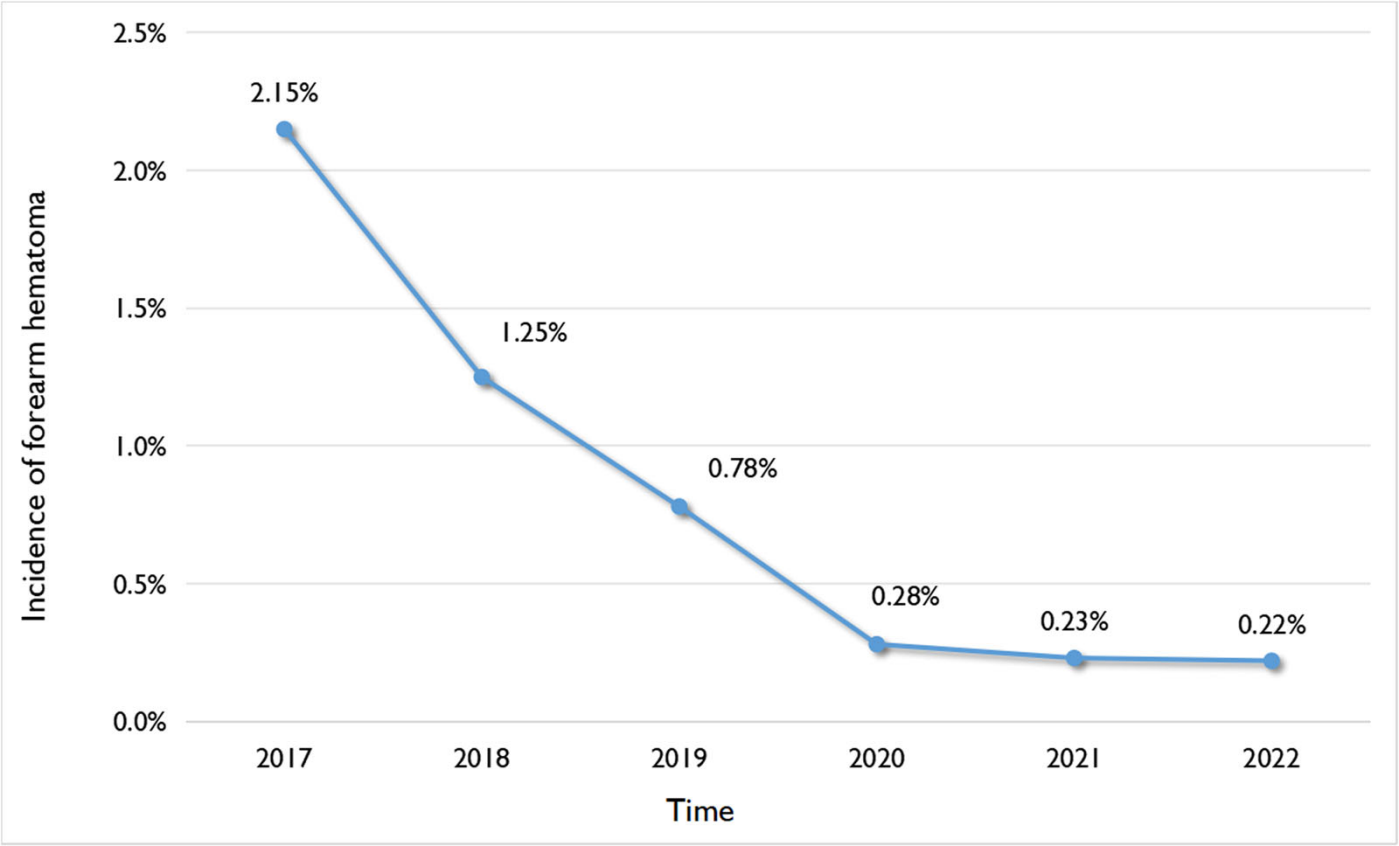
Incidence rate of forearm hematoma from 2017 to 2022.

We have calculated the incidence of forearm hematoma in our ward from 2017 to 2022, and it shows a decreasing trend year by year (see Figure 1). Now, we summarize our nursing experience of patients with forearm hematoma after transradial coronary intervention.

### 2.2 Development of grading criteria for forearm hematoma

We have recorded the occurrence and development of hematoma in all patients with forearm hematoma. On the basis of reviewing relevant literature and consulting experts, we have independently developed a grading standard for forearm hematoma, which involves multiple aspects such as hematoma range, pain grading, blisters, skin temperature, skin color, peripheral sensation, and pulse.

### 2.3 Analysis of typical cases

In this study, we selected three typical patients with forearm hematoma: mild hematoma, moderate hematoma, and severe hematoma. By observing, describing, and analyzing the pictures taken during clinical diagnosis and consultation, we summarize the prevention, treatment, and nursing of forearm hematoma after percutaneous coronary intervention, to provide reference for the nursing of patients with forearm hematoma. The patient pictures taken follow the principle of privacy protection. This research protocol complies with the guidelines of the Helsinki Declaration and has been approved by our institutional review committee and hospital ethics committee.

## 3 RESULTS

**Figure 2:**
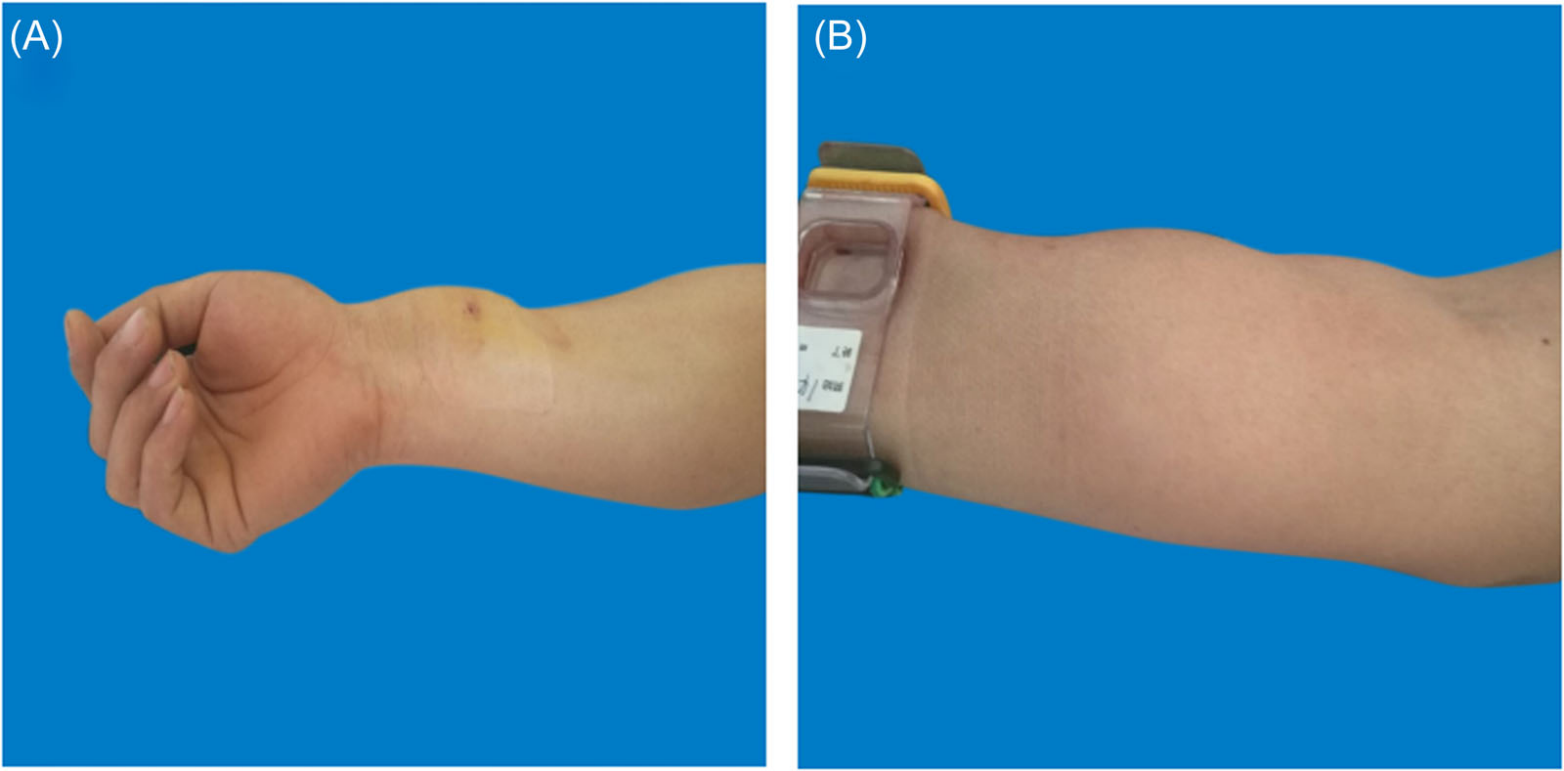
Mild Hematoma. A: The hematoma is located at the lower one‐third of the palmar side of the forearm, with a range of less than one‐third of the forearm. B: The hematoma is located at the middle one‐third of the palmar side of the forearm, with a range of less than one‐third of the forearm.

**Figure 3:**
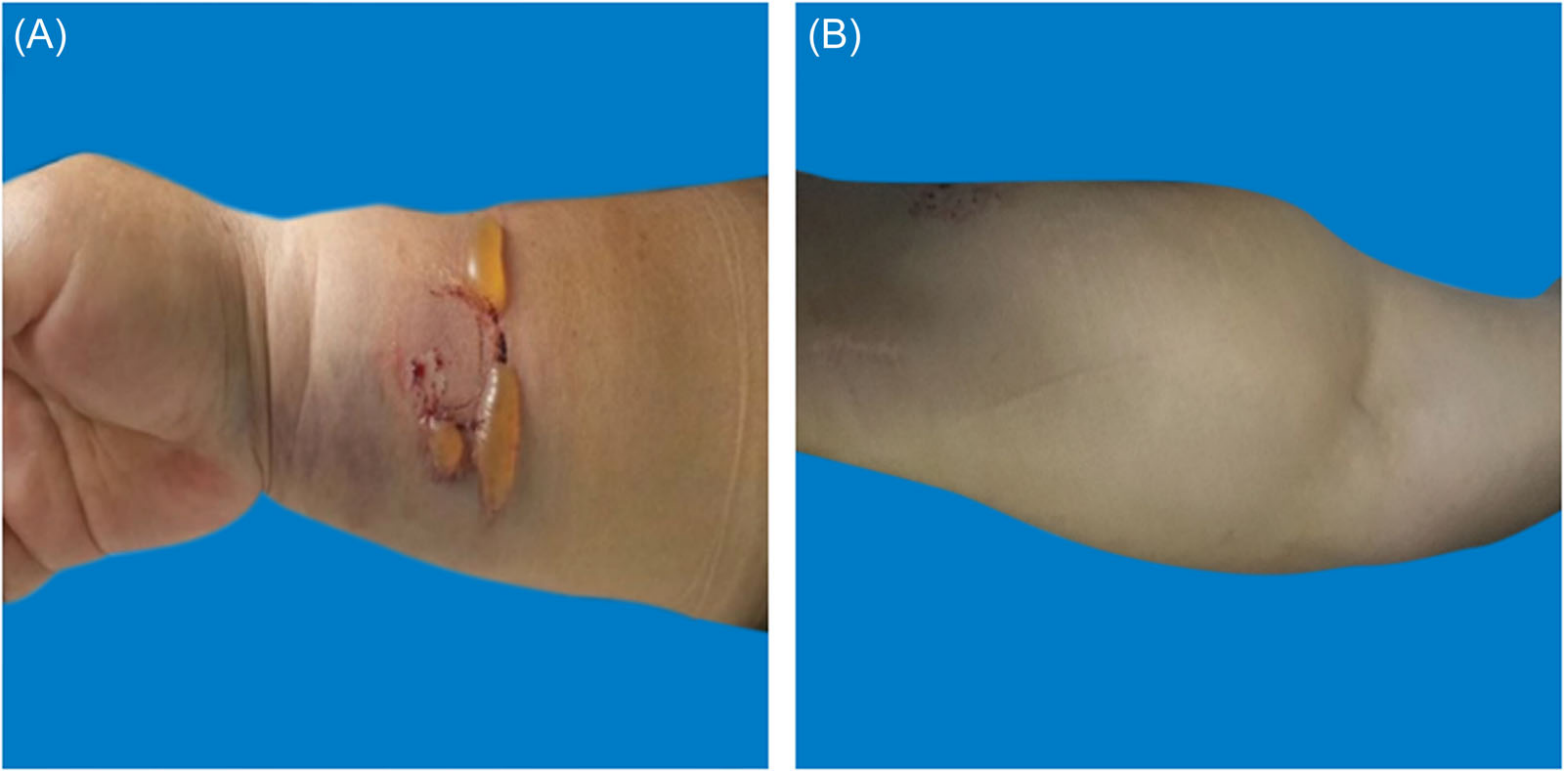
Moderate hematoma. A: The hematoma covers 1/3 to 1/2 of the forearm, with blisters. B: The hematoma covers 1/3 to 1/2 of the forearm, without blisters.

**Figure 4:**
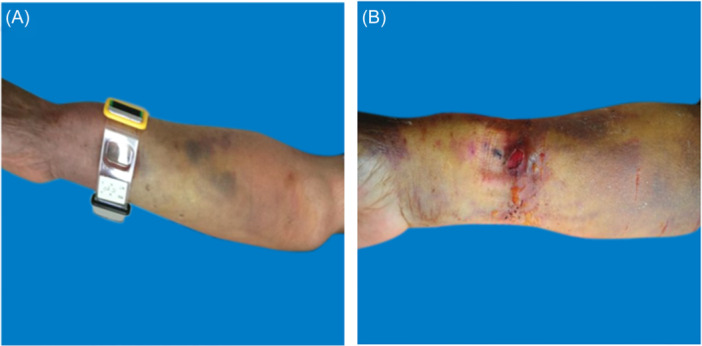
Severe hematoma. A: Hematoma extends beyond half of the forearm, accompanied by cyanosis. B: The hematoma extends beyond half of the forearm and the blister has ruptured.

On the basis of reviewing relevant literature and consulting experts, we have independently developed a grading standard for forearm hematoma, which involves multiple aspects such as hematoma range, pain grading, blisters, skin temperature, skin color, peripheral sensation, and pulse. It is possible to clearly distinguish different degrees of hematoma, and the standard is concise and easy to grasp (see Table 1). This study included three patients with forearm hematoma after transradial coronary intervention (1 males; 2 females). One female patient's hematoma was classified as mild hematoma (Figure 2), the puncture side limb was appropriately raised, compressed with an elastic bandage. and the puncture side limb was placed naturally. The elastic bandage was removed after 24 h of continuous use. During this period, nurses guided patients to engage in intermittent finger exercises； One male patient's hematoma was moderate hematoma (Figure 3), On the basis of appropriately raising the puncture side limb, elastic bandages were used to compress and bandage, and decompression was maintained 24 h later; The blister was punctured and the liquid can be sucked out. Wipe with physiological saline, and apply Hydrosorb Gel externally to promote wound recovery. The hematoma grading of another female patient was severe hematoma (Figure 4). Nurses paid attention to observing the skin temperature and color of the puncture side limb to maintain sufficient blood supply, as well as the pulsation of the radial artery on the puncture side. The patient has not implanted stents, the use of antiplatelet or anticoagulant drugs was temporarily suspended, and medication was resumed when bleeding stops and the hematoma is absorbed; Symptomatic treatments also was applied such as analgesics (non steroidal analgesics), Atrauman Ag. Three patients with forearm hematoma have recovered well and no related residual symptoms.

**Table 1:** Classification criteria for forearm hematoma.

Grade	Range	Pain^a^	Blister	Skin temperature	Color of skin	Peripheral sensation	Pulse
Mild Hematoma	The range of skin protrusions is less than one‐third of the forearm	Mild	No blisters	Normal	Visible bruising	Normal	Normal
Moderate hematoma	The range of skin protrusions reaches 1/3 to 1/2 of the forearm	Moderate	Occasional blisters	Rise	Bruising	Normal	Normal or slightly weak
Severe hematoma	The range of skin protrusions exceeds half of the forearm	Strong or worst possible	Blisters	Reduce	Cyanosis or pallor	Numb	Weakening or disappearing

^a^ Verbal Rating Scale (VRS): Pain intensity is divided into 5 levels: no pain, mild pain, moderate pain, strong pain, and worst possible pain.

**Figure 5:**
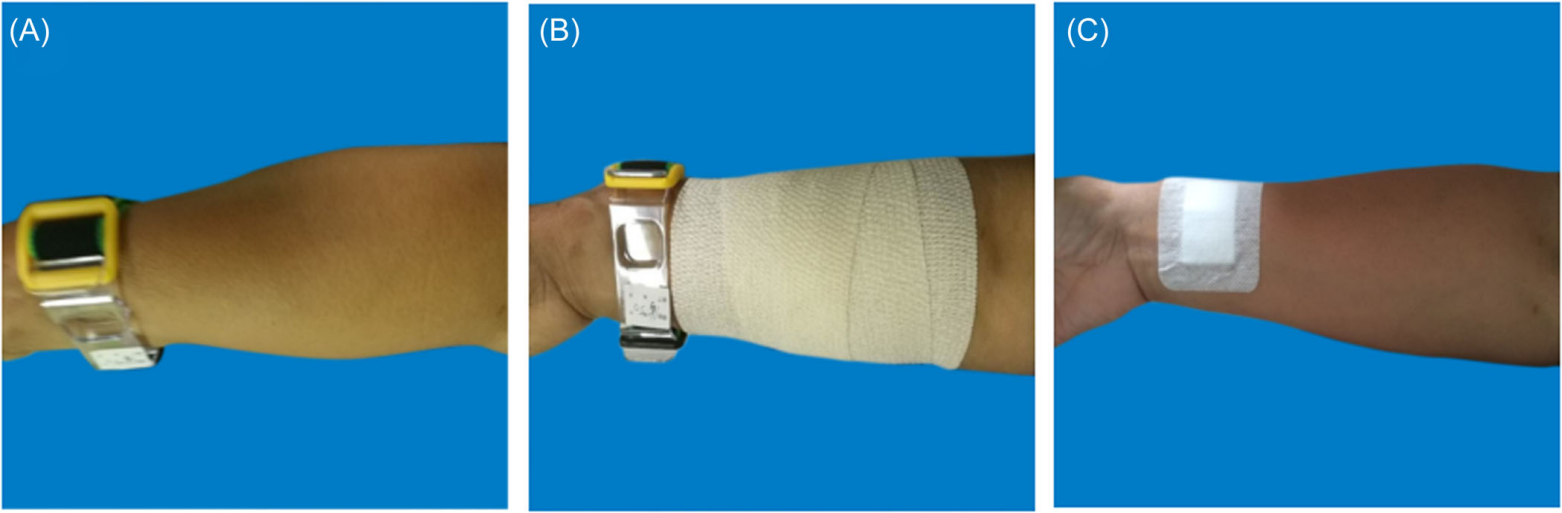
Treatment of mild hematoma. A: 0.5 h after percutaneous coronary intervention. B: Pressure bandage with elastic bandage. C: the next day after percutaneous coronary intervention.

**Figure 6:**
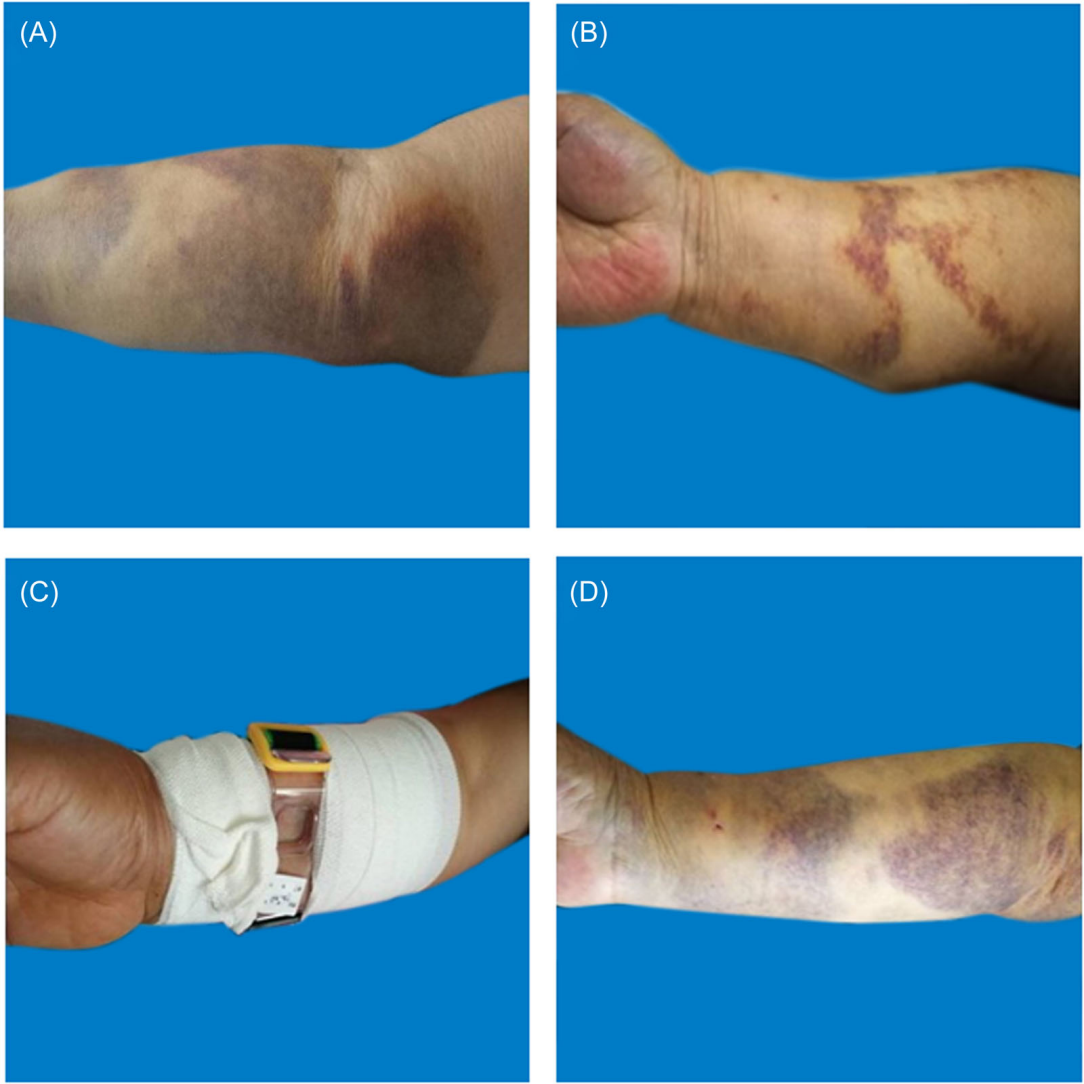
Treatment of moderate hematoma. A: Immediately after percutaneous coronary intervention. B: The third day after percutaneous coronary intervention. C: The first week after percutaneous coronary intervention. D: The third week after percutaneous coronary intervention.

**Figure 7:**
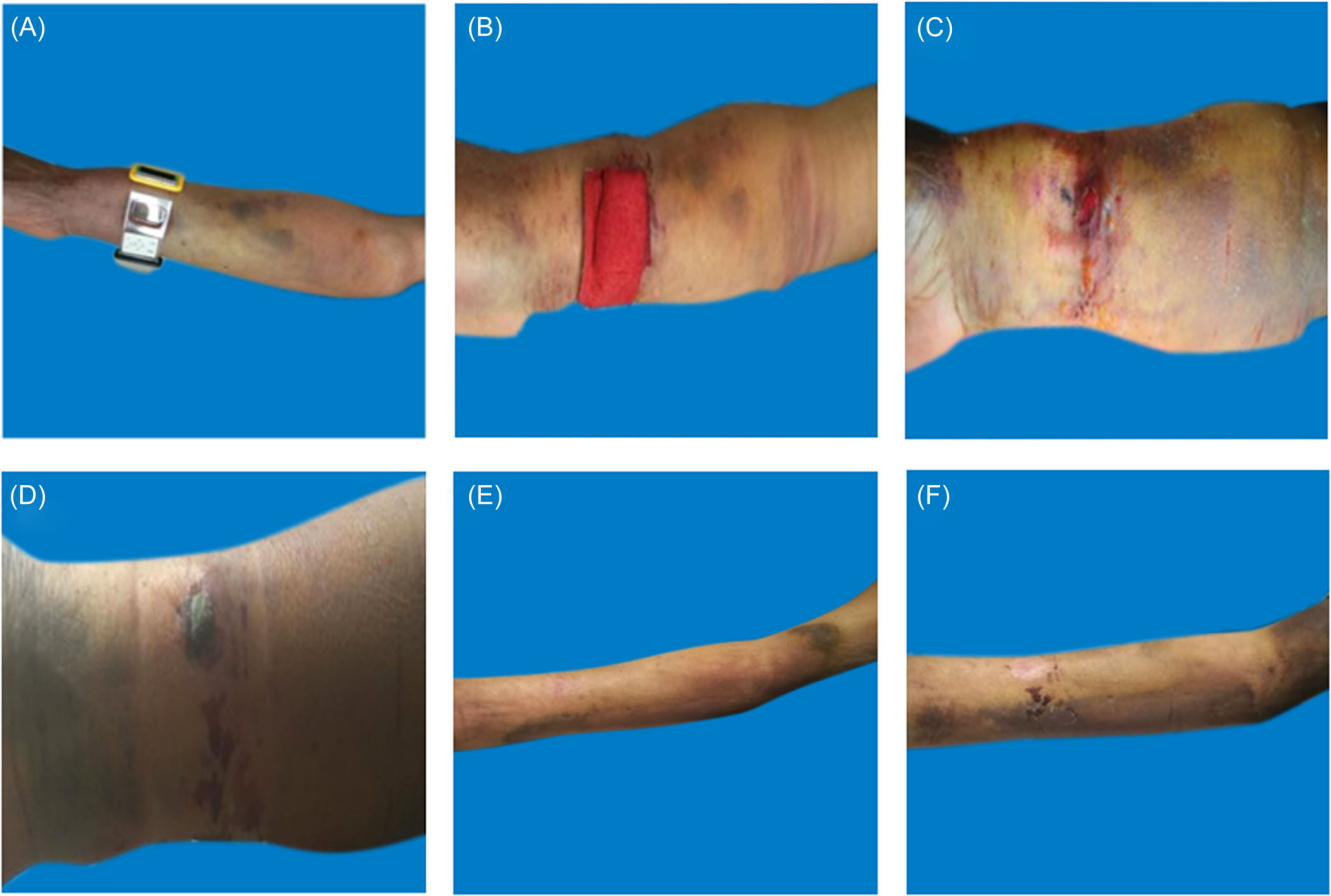
Treatment of severe hematoma. A: Immediately after percutaneous coronary intervention. B: When handing over to the ward. C: The next day after percutaneous coronary intervention. D: The eighth day after percutaneous coronary intervention. E:The third week after percutaneous coronary intervention. F:The sixth week after percutaneous coronary intervention.

## 4 DISCUSSION

### 4.1 Etiology

#### 4.1.1 Procedure factors

The key to successful transradial coronary intervention is for the operator to master the vascular anatomy of the right upper limb proficiently and possess skilled puncture techniques. Beginners may lack proficiency in puncture techniques, which may result in repeated or multiple punctures of the same site, or blind puncture damage to arterial vessels due to inaccurate determination of the position and direction of the radial artery. Due to the abundance of collateral vessels in the upper limbs, when a super smooth guide wire or catheter enters a small branch of the artery, it is easy to cause damage to the end of the branch; During the ascending process of the catheter, it may damage the blood vessel wall, causing bleeding, leading to the formation of a hematoma; Rough operation of guide wires and catheters can cause radial artery injury or even tearing. In addition, the inaccurate compression position of the compressor is also an important reason. Due to the fact that the puncture point of the skin and the blood vessel is not on the same vertical plane, if the compressor is placed too low to fully cover the puncture point of the blood vessel, it can lead to subcutaneous bleeding, forming a hematoma.

#### 4.1.2 Medical factors

Patients undergoing coronary intervention may require the use of anticoagulants or antiplatelet drugs, including use of aspirin, clopidogrel, ticagrelor, heparin, nadroparin, bivaludine, intravenous thrombolytic agents (urokinase, prourokinase, etc.), and IIb/IIIa receptor antagonists during the perioperative period, have a significantly increased incidence of forearm hematoma.^4^

#### 4.1.3 Patient factors

Patients with poor vascular conditions, such as small, tortuous, and deformed vascular lumens, can cause damage to vessels during the process of guide wire ascending; The patient experienced vascular spasm during PCI; Patients with elderly, female, elderly, low weight, or basic diseases such as hypertension, diabetes, liver and kidney dysfunction often have poor vascular conditions and high incidence of complications.

### 4.2 Risk classification of hematoma

Nurses need to pay attention to the grading of hematoma after PCI and provide personalized care based on different grades. At present, there are few grading standards for forearm hematoma after PCI, and the existing standards have certain shortcomings. Naveen Garg classified forearm hematoma into 5 levels: grade I, ＜5 cm in diameter (nonsignificant); grade II, 5‐10 cm diameter (mild); grade III, ＞10 cm but distal to the elbow (moderate); grade IV, extending above the elbow (severe); and grade V, anywhere with ischemic threat to the hand (compartment syndrome), mainly based on the swelling area as the grading standard, but did not refine other accompanying symptoms.^4^ Dangas only classified forearm hematoma into grade 2, including hematoma and major hematoma (hematoma + hematocrit drop＞15%), resulting in unsatisfactory differentiation of hematoma.^5^ On the basis of reviewing relevant literature and consulting experts, we have independently developed a grading standard for forearm hematoma, which involves multiple aspects such as hematoma range, pain grading, blisters, skin temperature, skin color, peripheral sensation, and pulse. The pain grading is based on verbal rating scale (VRS),^6^ and we have also conducted small‐scale validation of this hematoma classification standard, which involves comprehensive hematoma symptoms. It is possible to clearly distinguish different degrees of hematoma, and the standard is concise and easy to grasp (see Table 1). Due to the fact that the hematoma grading criteria used in this study cover multiple angles of evaluation, it has an early recognition effect on insufficient blood supply and nerve damage caused by excessive hematoma. Moreover, when combined with our hematoma management measures, this grading standard has exciting prognostic results. In the process of clinical application, we set different durations for elastic bandages based on the grading of the hematoma, as well as the timing for balancing with antiplatelet drugs. We determine whether blister treatment is necessary based on the diameter of the blister, and determine whether there is insufficient blood circulation and nerve damage based on pain grading, skin temperature, skin color, peripheral sensing, and pulse.

### 4.3 Treatment of hematoma

#### 4.3.1 Treatment of mild hematoma (Figure 5)

When the patient develops mild hematoma, the puncture side limb should be appropriately raised, compressed with an elastic bandage. and the puncture side limb should be placed naturally. The elastic bandage should be removed after 24 h of continuous use. During this period, nurses should guide patients to engage in intermittent finger exercises to promote blood circulation in the hands. Additionally, potato chips can be applied externally. If the patient has skin allergies or skin ulcers, potato chips are not recommended for use as they are prone to oxidation and are not recommended for long‐term use.

#### 4.3.2 Treatment of moderate hematoma (Figure 6)

When the patient develops moderate hematoma, on the basis of appropriately raising the puncture side limb, elastic bandages are used to compress and bandage, and decompression is maintained 24 h later; If accompanied by blisters, when the blister is greater than 1 cm, the blister can be punctured and the liquid can be sucked out. Wipe with physiological saline, and apply Hydrosorb Gel externally to promote wound recovery; When the blister is less than 1 cm, it should be closely observed without special treatment, and can be absorbed on its own.

#### 4.3.3 Treatment of severe hematoma (Figure 7)

When a patient develops severe hematoma, they should be bandaged with elastic bandages for 48‐72 h to maintain decompression. It is important to alternate between compression and decompression to maintain sufficient blood supply; Nurses should pay attention to observing the skin temperature and color of the puncture side limb, as well as the pulsation of the radial artery on the puncture side.

##### Other symptomatic treatments

In addition to local care of the hematoma, it is also necessary to cooperate with the doctor and adjust the patient's medication. The doctor decides whether to temporarily discontinue antiplatelet or anticoagulant drugs based on the patient's condition. If the patient has not implanted stents, the use of antiplatelet or anticoagulant drugs should be temporarily suspended, and medication should be resumed when bleeding stops and the hematoma is absorbed; However, for patients undergoing PCI, the risk of coronary ischemia is higher within 1 month after PCI. A comprehensive assessment of the patient's ischemic and bleeding risks is necessary to ultimately determine whether the patient needs to discontinue antiplatelet or anticoagulant therapy. Symptomatic treatments such as analgesics (non steroidal analgesics), dehydrating agents (mannitol injection), Hydrosorb Gel, Atrauman Ag, Mucopolysaccharide Polysulfate Cream (Hirudoid) and other topical applications can also be applied appropriately.

#### 4.3.4 Choose the appropriate elastic bandage for compression wrapping

Tight elastic bandages can cause obstruction of venous blood flow; If it lasts for a long time, it may cause a slowdown in the blood flow, and the nerve endings become sluggish due to a lack of oxygen supply, further leading to numbness. The pressure of the elastic bandage should be maintained within the range of palpable fluctuations in the radial artery of the puncture side limb, while paying attention to any ischemic symptoms such as pain, numbness, and abnormal activity. Regarding the selection of elastic bandages, research has shown that Idealast‐haft elastic bandages have greater advantages in the treatment of forearm hematoma compared to Nylexorgrip elastic bandages.^3^ Idealast‐haft elastic bandages has characteristics such as short ductility, high cotton content, no latex, firm fabric structure, long‐lasting elasticity, self‐adhesive, and not easy to loosen. Therefore, Idealast‐haft elastic bandages are commonly used in our department.

#### 4.3.5 Other treatment measures

Other treatment measures include: applying ice to the hematoma within 24 h can reduce the severity of the hematoma, and after 24 h, methods such as towel hot compress and infrared therapy can be used; Quiet infusion of papaverine; Blood pressure cuff compression hemostasis; Local wet compress of traditional Chinese medicine; Incision and drainage, etc.

### 4.4 Prevention

#### 4.4.1 Preoperative evaluation

Transradial coronary intervention requires the radial artery to be thick, straight, and elastic, while also considering the compensatory function of the ulnar artery. Before PCI, the condition of hand vessels should be assessed, and the clinical routine should use the modified Allen test or blood oxygen saturation detection method;^7^, ^8^ However, there are many influencing factors for both methods, including whether radial artery compression is complete, patient skin color and light, which can cause misjudgment. We jointly use B‐ultrasound to detect radial artery blood flow and exclude the influence of the above factors. On the day before PCI, color doppler ultrasound is applied to evaluate the diameter, wall thickness, and to monitor the blood flow velocity and direction of the radial and ulnar arteries. If necessary, the left radial artery or right femoral artery approach can be considered.

#### 4.4.2 Intraoperative operations

When performing arterial puncture, choose the appropriate puncture site, comfort the patient in advance, strive for successful first puncture, and avoid repeated punctures; During the puncture process, the operation should be gentle; Puncturers should always maintain a clear mind, move forward and backward in a timely manner, and take emergency measures.

#### 4.4.3 Postoperative observation

Early observation of hematoma and timely compression treatment are very important. During the process of returning from the catheterization room to the ward after PCI, patients should pay attention to the dynamic observation of the “three time points”. Firstly, at the end of the PCI, the operator should pay attention to whether there is swelling around the puncture site when applying pressure to stop bleeding for the patient; Secondly, when a patient is transferred from the buffer room to the ward, the doctors and nurses in the buffer room should pay attention to the condition of the patient's puncture site. If there is bleeding or swelling, it should be dealt with in a timely manner; Thirdly, after the patient returns to the ward, the ward nurse should first observe the puncture site, measure the arm circumference of the upper and lower edges of the compressor, record the values in detail, measure every hour, and observe for bleeding and swelling. It is emphasized that the observation of the entire puncture limb should not be limited to the puncture site.

#### 4.4.4 Postoperative evaluation and education

**Figure 8:**
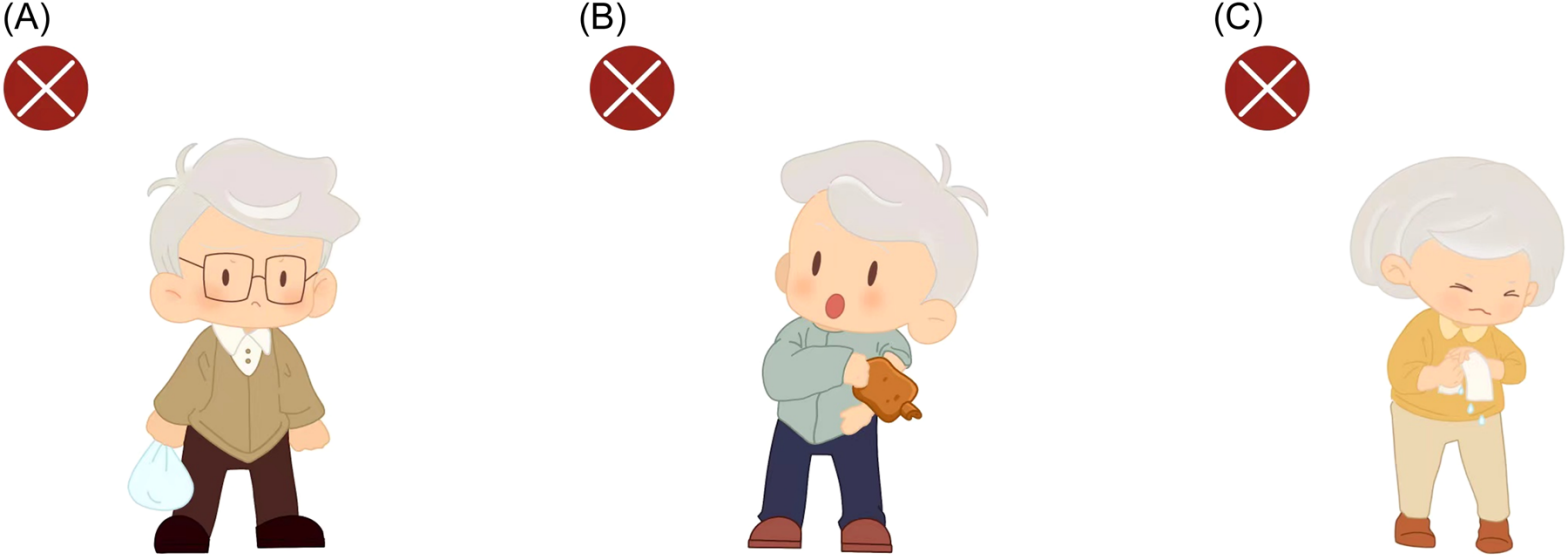
Activity guidance for puncturing limbs. A: Not to bear weight on the puncture limbs. B: Not to apply hot compress on the puncture limbs. C: Not to twist towels.

After surgery, nurses each shift evaluates the puncture site for bleeding and hematoma, observes whether the dressing is clean, dry, and fixed in place, evaluates the temperature, color, and sensation of the punctured limb, and keeps records. When evaluating arterial pulsation, nurses need to pay attention to the “5 P syndrome”, including pain, pallor, pulseless, paresthesia, and paralysis.^9^ If these indicators are abnormal, it indicates a certain degree of ischemia and should be reported to the doctor in a timely manner. In addition, nurses should provide comprehensive discharge education to patients: if patients need to use anticoagulants, they should be informed to strengthen their observation of bleeding. If there is gum bleeding, vomiting blood, hematuria, black stool, or skin bleeding, they should immediately contact a doctor for follow‐up. Nurses should inform patients not to bear weight on the puncture limbs, not to apply hot compress, and not to twist towels within 30 days after PCI (Figure 8).

## 5 CONCLUSION

Forearm hematoma after transradial coronary intervention is the most common complication, especially for beginners and medical institutions that have just started coronary intervention. By identifying risk factors and mastering management measures, the incidence of forearm hematoma after intervention can be effectively reduced, and patient comfort can be improved. Meanwhile, strengthening preoperative evaluation, standardized intraoperative procedures, postoperative care, and rational drug treatment are key measures for prevention. In the future, intelligent monitoring devices can be used for real‐time monitoring and pressure adjustment of forearm hematoma to improve the timeliness and accuracy of hematoma observation. Remote radial artery puncture technology is considered to continuously improve nursing quality and level.

## INFORMED CONSENT DECLARATION

We fully informed the patients involved in this study that they agreed to participate and signed an informed consent form.

## STATEMENT OF NON‐DUPLICATION

We certify that our manuscript is a unique submission and is not being considered for publication by any other source in any medium.

## AUTHOR CONTRIBUTIONS

**Guangshuo Zhi**: Data curation; Writing—original draft. **Mengjie Lei**: Writing—original draft. **Shuang Qian**: Data curation. **Chunyan Zhang**: Data curation. **Yachao Li**: Writing—review and editing. **Zhigang Zhao**: Writing—review and editing. **Zengming Xue**: Writing—review and editing; Supervision; Conceptualization.

## CONFLICT OF INTEREST STATEMENT

There are no conflicts of interest in this manuscript. The fund projects involved in this manuscript are sourced from the government and do not involve any conflicts of interest.

## TRANSPARENCY STATEMENT

The lead author Zengming Xue affirms that this manuscript is an honest, accurate, and transparent account of the study being reported; that no important aspects of the study have been omitted; and that any discrepancies from the study as planned (and, if relevant, registered) have been explained.

## Data Availability

All the data generated or analyzed during this study are included in this article and/or its supplementary material files. Further enquiries can be directed to the corresponding author. All data generated or analyzed during this study are included in this published article [and its supplementary information files].
